# Human Herpesvirus-6A and -6B (HHV-6A and HHV-6B): The Role of Roseoloviruses in Neurological Dysfunction and the Mechanisms of Viral-Induced Epileptogenesis

**DOI:** 10.3390/v18060660

**Published:** 2026-06-10

**Authors:** Elham Bahramian, Ananya Bajpai, Xue Yang, Dana M. Cairns, David Kaplan, Ruben M. Ceballos

**Affiliations:** 1 Molecular and Cell Biology Department, University of California Merced, Merced, CA 95343, USA; 2 Host-Virus Evolutionary Dynamics Institute, University of California Merced, Merced, CA 95343, USA; 3 Quantitative Systems Biology Program, University of California Merced, Merced, CA 95343, USA; 4 Department of Biomedical Engineering, Tufts University, Medford, MA 02155, USA; 5 Center for Interdisciplinary Neuroscience, University of California Merced, Merced, CA 95343, USA; 6 Health Science Research Institute, University of California Merced, Merced, CA 95343, USA

**Keywords:** herpesvirus, roseolovirus, HHV-6, epilepsy, neuropathology, viral disease

## Abstract

Human herpesvirus-6 consists of a pair of viral species, HHV-6A and HHV-6B, which are neurotropic with the ability to invade, persist, and reactivate within the nervous system. Accumulating evidence links HHV-6 to epilepsy and other neuropathologies, including: multiple sclerosis, chronic fatigue syndrome, and neurodegeneration. Yet, mechanisms by which these viruses induce neurological disorders, including their role in epileptogenesis, remain unknown. It has been demonstrated that HHV-6 exhibits tropism for astrocytes, oligodendrocytes, and neurons. Thus, HHV-6 can perturb cellular homeostasis, neuronal signaling, and immune regulation, astrocytic glutamate clearance, GABAergic inhibition, and cholinergic or monoaminergic neurotransmission yielding network hyperexcitability. It is also reported that HHV-6 can activate neuroinflammation through Toll-Like Receptor (TLR), cytokine, and/or NF-κB activation, which facilitates neuronal injury and network instability. Indeed, a suite of converging processes suggest a multifactorial nature for HHV-6 related neuropathology. Despite robust experimental and clinical data, definitive causal relationships between HHV-6 and epilepsy (or induction of neurodegeneration) remain elusive. This review discusses evidence for roseolovirus-induced neurological dysfunction and disorders commonly associated with HHV-6A and HHV-6B infections. A preponderance of clinical and experimental evidence suggests that differential tropism for distinct neuronal neurotransmitter chemotypes and glia as well as systemic effects are involved in roseolovirus-mediated neurological disease.

## 1. Introduction

Roseolovirus infections may induce or exacerbate a wide array of neuropathologies. Although mechanisms for virus-mediated neurological dysfunction for several disorders are debated—including: chronic fatigue syndrome (CFS); neurodegenerative disorders (NDD); and epilepsy [1,2,3,4]—the etiologies of other diseases, such as multiple sclerosis (MS), are well-studied [5,6,7,8].

With regard to epilepsy, neurotropic viral infection is decidedly acknowledged as a viable trigger for isolated seizure events as well as a precursor to chronic seizure activity. Seizure induction can occur either through acute neuro-encephalitis or lasting molecular and cellular changes that persist after viral latency is established [9,10]. Among the viruses known to induce seizure events are the roseoloviruses, specifically, human herpesviruses 6A and 6B (HHV-6A and HHV-6B). These two viruses have emerged as suspected etiologic agents capable of inducing seizures and epilepsy. Due to high seroprevalence in humans (~90% of the population is seropositive), their ability to infect cells of the central nervous system (CNS), and their capacity to establish lifelong chronic infections in neural tissues, these roseoloviruses are principal suspects in virus-induced epileptogenesis and several other neurological and neurodegenerative disorders [11,12,13,14,15]. HHV-6A and HHV-6B are two closely related viruses that are recognized as distinct viral “species” rather than simply two strains of the same virus. This is justified since the viruses differ in: cell tropism, relative virulence on select permissive host cell types, receptor usage, and associations with different diseases [16,17,18,19]. While HHV-6B is commonly associated with childhood infection and febrile seizures, both HHV-6A and HHV-6B have been detected in adult brain tissue and implicated in chronic neurological dysfunction [15,20,21].

Epilepsy is among the most prevalent neurological disorders worldwide and remains a major clinical challenge due to its heterogeneous etiologies and frequent resistance to available anti-seizure therapies. Temporal lobe epilepsy (TLE), the most common focal epilepsy in adults, is particularly refractory to treatment and is commonly associated with progressive structural and functional alterations within limbic brain regions, including the hippocampus, amygdala, and entorhinal cortex [9,22]. Although there are certainly genetic factors and developmental abnormalities that can contribute to TLE pathogenesis, experimental and clinical evidence also demonstrates that acquired insults including viral infections of the central nervous system (CNS) play a critical role in initiating and shaping epileptogenic processes [9,10,23,24].

For example, DNA, RNA, and viral antigens of both HHV-6A and HHV-6B have been detected at higher frequency in resected brain specimens from patients with mesial temporal lobe epilepsy (MTLE) compared to non-MTLE controls, particularly within the hippocampus and amygdala, which are regions of noted importance in seizure generation and propagation [25,26,27]. Immunohistochemical and molecular analyses also show that HHV-6A and HHV-6B readily infect astrocytes, although infection of oligodendrocytes and neurons is also documented [28,29]. Despite observed correlations between presence of HHV-6 molecular markers and neurological diseases, causal relationships between HHV-6 infection and epilepsy remain debated. Because roseolovirus DNA may also be found in individuals without epilepsy and chromosomally integrated HHV-6 provirus can complicate the interpretation of viral load measurements [30,31,32,33], “cause-and-effect” relationships are difficult to discern. Nevertheless, accumulating evidence supports the hypothesis that persistent or reactivated HHV-6B infection can contribute to epilepsy by altering glial function, neurotransmitter homeostasis, and neuroimmune signaling [27].

In this review, we offer an overview of roseolovirus biology and pathogenic potential as well as current evidence linking HHV-6A and HHV-6B to neurological dysfunction and disease. This is followed by a description of the best-studied mechanistic links between roseolovirus infection and neurological dysfunction [4,15,21]. The remainder of this review is then dedicated to describing how HHV-6 may induce seizure and contribute to epileptogenesis with a focus on temporal lobe epilepsy (TLE). This includes a description of the five current hypotheses regarding HHV-6-induced epileptogenesis. Since HHV-6 may also impact cholinergic transmission, we include nascent data regarding the potential role of HHV-6 in neurodegeneration. Lastly, we review experimental strategies to test select hypotheses regarding roseolovirus-induced neurological dysfunction.

## 2. Human Herpesvirus-6 (HHV-6) Biology and Pathogenic Potential

HHV-6 was first isolated in 1986 from patients with lymphoproliferative disorders and AIDS. The virus was initially designated human B-lymphotropic virus (HBLV) [11]. Shortly thereafter, additional isolates were independently identified in HIV/AIDS patients from Africa, including the U1102 and Z29 strains. Further studies revealed that HHV-6 is a widely distributed human herpesvirus [12,13]. Subsequent molecular, immunological, and epidemiological analyses demonstrated that HHV-6 comprises two genetically and biologically distinct viruses, HHV-6A and HHV-6B, which differ in genomic organization, antigenic properties, cell tropism, and disease associations [14,16,34,35]. The distinctions are not merely taxonomic but have important implications for viral persistence, immune modulation, and neuropathogenic potential.

HHV-6 belongs to the *Betaherpesvirinae* subfamily of the virus family *Herpesviridae*, which reflects the genetic relationships between HHV-6A, HHV-6B, HHV-7, and HHV-5 (known as human cytomegalovirus) [36]. Genomic sequencing confirms that HHV-6A and HHV-6B share substantial sequence homology with one another while retaining species-specific differences in regulatory regions, glycoproteins, and immediate early genes that influence viral replication and host interactions [17,18,19,37,38]. Both viruses have a double-stranded (ds) DNA genome of ~160 kb with unique regions flanked by direct repeat sequences (DRL and DRR), which contain viral packaging signals and notable telomere-like repeats [30,38]. The telomeric motifs enable site-specific integration of the viral genome into the telomeres of the human chromosome, a distinctive feature of HHV-6 that contributes to lifelong persistence and the potential for reactivation [31,32,39,40].

Integration of HHV-6 into germ cells results in inherited chromosomally integrated HHV-6 (iciHHV-6; a.k.a., eHHV-6), which is present in ~1% of the human population [31]. The HHV-6 U94 gene product (pU94) is decidedly related to a protein found in parvovirus. The sequence identity (~24%) between pU94 and adeno-associated virus type 2 (AAV-2) Rep68/78 protein is notable. Since Rep family activity includes DNA binding at telomeric repeats as well as ATPase, helicase, and exonuclease activity [41,42], pU94 may facilitate HHV-6 telomeric integration. Also, T cells that stably express pU94 are refractory to lysis and exogenous application of pU94 blocks HHV-6 replication in a dose-dependent manner, suggesting a potential role for pU94 in latency and persistence in host tissues.

Despite overall genomic sequence identity (~90%), HHV-6A and HHV-6B exhibit marked divergence in specific genomic regions, which in turn influences viral dynamics. The direct repeats and the right end of the unique region of ORFs U86–U100 are among the most variable segments between the two viruses [40]. The immediate-early 1 (IE1) proteins encoded by U89–U09 show limited homology between the two viruses, resulting in differences in transcriptional regulation and immune function [41,43]. Variability in glycoproteins such as gB, gH, and components of the gH, gL, and gO complexes further contributes to differences in viral attachment and entry and cell tropism [44,45,46,47]. These distinctions are considered determinants of virus-specific disease associations.

In general, HHV-6 exhibits broad cell tropism, replicating in CD4^+^ T lymphocytes, monocytes, macrophages, dendritic cells, fibroblasts, and natural killer cells [48,49,50,51,52,53,54,55,56,57,58]. HHV-6 is also shown to infect oligodendrocytes, astrocytes, and microglia (Table 1) as well as vascular endothelial cells and different types of neurons (Table 2). The cluster of differentiation factor 46 (i.e., CD46) was considered the principal cell surface protein used by HHV-6 as a receptor for attachment and entry; however, it was later reported that there is differential affinity for CD46 between HHV-6A versus HHV-6B. Indeed, CD134 has been identified as a preferred cell surface receptor for HHV-6B [59,60,61]. Current consensus is that HHV-6A primarily uses CD46 while HHV-6B preferentially uses CD134, a receptor largely restricted to activated T lymphocytes [59,60,61]. Consequently, non-specific receptor usage of HHV-6B may contribute to the broad cell tropism of this virus.

The ability of HHV-6 to infect both immune and neural cell populations positions the virus as an etiological agent for diseases that intersect immune dysregulation and neural dysfunction. Beyond the impacts of progeny virion production, HHV-6 exerts profound immunomodulatory effects that shape host responses. HHV-6 infection alters cytokine production, interferon signaling, and natural killer cell activity, while inducing apoptosis of infected T cells both in vitro and in vivo [6,72,73,74,75]. HHV-6 genomes encode multiple immunoregulatory proteins, including chemokine receptor homologs (U12 and U51) and viral chemokines such as U83 [7,65]. Indeed, these differ between HHV-6A and HHV-6B, resulting in virus-specific recruitment of select immune cell subsets [7,65]. As a result, the ability for different gene expression strategies enables HHV-6 to evade immune clearance, expand host cell range, and sustain low-level persistence.

Epidemiologically, HHV-6B is considered a common viral pathogen in children and acts as an etiologic agent of roseola infantum (a.k.a., exanthema subitem or sixth disease), typically a benign illness that manifests as a transient rash on the trunk and or limbs with or without fever. However, infection can be complicated by inducing febrile seizures [76]. In contrast, primary HHV-6 infection in adults is less common and may manifest as a mononucleosis-like syndrome [77]. 

Although HHV-6A infection was initially considered less prevalent, improved serological discrimination has revealed that this virus is also widely distributed and frequently associated with chronic inflammatory and neurological ailments [78,79]. Reactivation of either of the viruses, especially in immunocompromised individuals, has been linked to neuro-encephalitis, seizures, cognitive dysfunction, and other neurological manifestations [15,33,42,80].

Cumulatively, properties of HHV-6 infection—including: a capacity for chromosomal integration; broad cell tropism; immune modulation; and species-specific pathogenesis—offer a variety of mechanisms by which the viruses may induce or exacerbate neurological disorders and thus contribute to central nervous system dysfunction.

**Figure 1:**
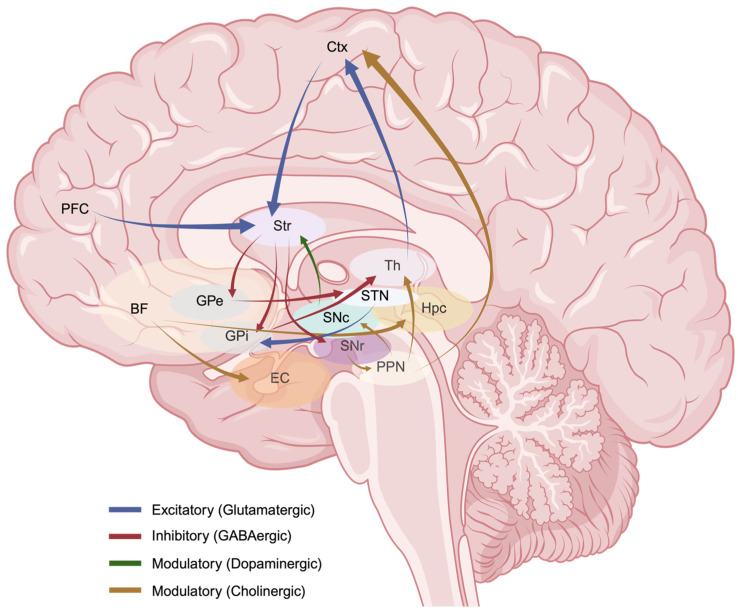
Glutamatergic, GABAergic, cholinergic, and dopaminergic connections in the brain. Schematic representation of glutamatergic (blue), GABAergic (red), dopaminergic (green), and cholinergic (gold) connections linking: cortex (Ctx), prefrontal cortex (PFC), striatum (Str), thalamus (Th), substantia nigra pars compacta (SNc), substantia nigra pars reticulata (SNr), globus pallidus externa (GPe), globus pallidus interna (GPi), subthalamic nucleus (STN), basal forebrain (BF), entorhinal cortex (EC), hippocampus (Hpc), and pedunculopontine nucleus (PPN)—via excitatory, inhibitory, and modulatory pathways.

Parsing the relative contribution of each virus to a myriad of pathways (Figure 1) that lead to neurological dysfunction and associating individual, additive, and synergistic mechanisms to specific diseases has proven challenging. However, an examination of disruptions to cell function due to virus infection can provide insights into the potential role of roseolovirus-induced epilepsy. Specifically, if nerve cell dysfunction can be related to neural network signal disruptions in glutamatergic, GABAergic, cholinergic, and/or monoaminergic pathways, a better understanding of roseolovirus-driven epileptogenesis may be ascertained. In the following sections, we tease out relationships between roseolovirus infection and specific virus–host interaction dynamics to address this topic with a focus on TLE.

## 3. Neurological Disorders Associated with Roseoloviruses

HHV-6 is a neurotropic virus capable of infecting and persisting within the central nervous system (CNS). Both HHV-6A and HHV-6B have been detected in brain tissues (Table 3), cerebrospinal fluid (CSF), and peripheral compartments of patients suffering from neurological disease, demonstrating the ability of roseoloviruses to cross the blood–brain barrier and establish long-term latency within neural tissue [5,81,82]. In this section, we review the features of the roseolovirus replication cycle, discussing neurotropism and cell dysfunction leading to inflammation, neurodegeneration, and common neurological disorders associated with active HHV-6 infection.

### 3.1. Central Nervous System Neurotropism and Cellular Targets

Viral entry of HHV-6 into the CNS occurs during primary viremia or via retrograde neuronal transport, which results in active and/or latent infections in the hippocampus, amygdala, olfactory pathways, and other areas of the brain [25,84].

At the cellular level, HHV-6 exhibits broad tropism in the CNS. Electron microscopy and histopathological studies have revealed infection of oligodendrocytes by HHV-6B, with additional evidence supporting infection of astrocytes and microglial cells in vivo [28,85,86]. Immunohistochemical analyses of resected tissues from TLE patients resolve HHV-6 antigens in cells expressing glial fibrillary acidic protein (GFAP), thus implicating astrocytes as a major viral reservoir in epileptogenic brain regions [25]. In vitro studies confirm that both HHV-6A and HHV-6B readily infect astrocytes, leading to functional alterations in glutamate uptake and inflammatory signaling [29].

More recent work has expanded the cellular spectrum of HHV-6 infection to include neurons. HHV-6A and HHV-6B have been shown to infect Purkinje cells [71], and our lab has demonstrated that both roseoloviruses infect human glial cells and neurons, including glutamatergic and dopaminergic neurons differentiated from human neural stem cells [67]. Notably, we were unable to detect HHV-6A or HHV-6B infection in GAD67+ cells (i.e., GABAergic cells) [67]. Selective neuronal tropism shown in vivo would provide a framework for explaining how HHV-6 infection may disrupt excitatory versus inhibitory circuits within the CNS, leading to epileptiform activity. Susceptibility of ChAT+ and AChE+ cells would implicate infection of cholinergic neurons as an additional potential factor in epileptogenesis and, possibly, neurodegeneration related to Alzheimer’s disease (AD). Whether susceptibility of distinct neuronal chemotypes is a function of CD46 or CD134 expression is unclear; however, understanding differential cell tropism between HHV-6A and HHV-6B is a critical component to understanding roseolovirus-mediated neurological dysfunction.

Accumulating clinical and experimental evidence has linked HHV-6 infection to a spectrum of neurological disorders. HHV-6B is most consistently associated with febrile seizures, encephalitis, and epilepsy—particularly temporal lobe epilepsy [87]. In contrast, HHV-6A is frequently detected in patients with chronic neuroinflammatory conditions, including multiple sclerosis (MS), where roseolovirus DNA, mRNA, and antigens have been detected in blood, CSF, and CNS tissue [5,6,7,8,74]. Comparative studies examining HHV-6A versus HHV-6B prevalence in neurological disease suggest species-specific pathogenic tendencies. HHV-6A DNA and transcripts are more commonly detected in patients with inflammatory demyelinating disorders, while HHV-6B presents a stronger association with seizure-related neuropathologies [20,53,88]. HHV-6A infection has been identified within the blood and CSF of patients with relapsing–remitting MS (RRMS), and viral localization in CNS tissue has been reported in a subset of affected individuals [5,6]. In pediatric oncology, HHV-6A has been detected in up to 72% of glial tumors, further supporting its neurotropic and potentially oncogenic capacity [75].

As stated above, receptor usage and cell tropism are likely contributors to distinct disease associations. Although both HHV-6A and HHV-6B bind CD46, they appear to bind distinct CD46 isoforms for viral attachment and entry [72,89]. Moreover, HHV-6B will additionally bind to CD134 and at higher affinity than with CD46. CD134 is expressed primarily on activated T lymphocytes. Thus, HHV-6B use of CD134 may influence viral dissemination and immune function during infection [60]. Such differences in receptor use ultimately shape cell tropism, viral persistence, and the downstream neuroimmune effects of roseolovirus infections.

### 3.2. Chronic Neuroinflammation and Neurodegeneration

Beyond acute neurological syndromes, HHV-6 has been implicated in generalized neuroinflammation and neurodegeneration. In multiple sclerosis (MS), HHV-6A has been detected in lesions, plaques, and otherwise normal-appearing white matter (Table 4). HHV-6A viral antigens have also been localized to both oligodendrocytes and astrocytes, suggesting a potential role in demyelination and chronic neuroinflammation [5,6,7,8,74].

Emerging evidence also implicates HHV-6 in dementia and Alzheimer’s disease (AD). Neurodegenerative disorders such as AD are characterized by neuroinflammation, immune dysregulation, and accumulation of pathological protein aggregates (NPP). Viral infections, including herpesviruses, are proposed as external modulators of amyloid-beta (Aβ) dynamics and inflammatory signaling within the brain. Recent studies demonstrate that HHV-6A infection can induce Aβ expression and activate microglia, supporting a link between roseolovirus infection, neuroinflammation, and neurodegeneration [2]. In addition to independent effects, HHV-6 can trigger reactivation of other latent viruses such as Epstein–Barr virus (EBV) [90], cytomegalovirus (CMV) [91], Varicella Zoster virus (VZV) and herpes simplex virus 1 (HSV-1) [92], which are also implicated in AD and MS pathogenesis. HHV-6 may exert neurodegenerative effects directly or indirectly through reactivation of these other viral mediators [64]. These observations align with the broader hypothesis suggesting that herpesvirus infection will accelerate amyloid deposition and neuronal injury through inflammatory and immune-mediated pathways.

### 3.3. HHV-6 in Myalgic Encephalomyelitis/Chronic Fatigue Syndrome (ME/CFS)

HHV-6 infection has also been investigated as a putative etiologic agent in the onset or complications of myalgic encephalomyelitis/chronic fatigue syndrome (ME/CFS), a neurological disorder characterized by profound fatigue, neurocognitive dysfunction, and immune system abnormalities. HHV-6 establishes lifelong latency and can undergo periodic reactivation, making it a plausible contributor to chronic multi-system diseases [21,93].

Several studies suggest associations between HHV-6 reactivation and mitochondrial dysfunction, metabolic reprogramming, and immune dysregulation in ME/CFS patients. Reactivation of HHV-6A or HHV-6B is shown to induce mitochondrial fragmentation and alterations in host cell metabolism, including a significant upregulation of one-carbon metabolic enzymes and a significant suppression of oxidative phosphorylation [94,95]. Soluble factors released from cells infected with HHV-6 can induce a sustained antiviral danger response in uninfected cells, suggesting a paracrine signaling mechanism during systemic dysfunction [48].

Post-mortem studies further support direct involvement of viral infection in ME/CFS. Specifically, widespread HHV-6 activation has been shown across multiple brain regions in ME/CFS patients but not in controls [96]. Epidemiological studies report high frequency of HHV-6A detection in ME/CFS cohorts compared to healthy individuals; however, HHV-6B prevalence appears similar between groups [1]. More recent cohort studies show that persistent HHV-6A and HHV-6B infection correlates with elevated pro-inflammatory cytokines (Table 5) as well as immune-related receptors, immune-related growth factors, and other molecules (Table 6) in patients with ME/CFS and a suite of other neurological diseases, reinforcing a role for viral reactivation in disease severity [97,98].

### 3.4. HHV-6 and Multiple Sclerosis

Multiple sclerosis (MS) is a chronic autoimmune demyelinating disease of the central nervous system (CNS) characterized by focal inflammatory lesions within the brain and spinal cord, leading to impaired signal conduction along axons and progressive neurological dysfunction [103,104]. MS has been clinically classified into four defined phenotypes: relapsing-remitting MS (RRMS); clinically isolated syndrome (or progressive relapsing); primary progressive MS (PPMS); and secondary progressive MS (SPMS). Across all four phenotypes, there are ~2.3 million people worldwide suffering from MS with initial onset manifesting in young adults between 20 and 40 years of age [105]. Prevalence in females is higher than in males at a ratio of ~3:1 [105]. Among the MS phenotypes, patterns of relapse and disability progression vary [106].

A majority of patients initially present with CIS or RRMS, which are characterized by episodic neurological deficits lasting days to weeks that may include cognitive symptoms (e.g., disorientation) as well as physical symptoms (e.g., numbness).

Episodes are separated by periods of partial or seemingly complete recovery. However, many patients will ultimately transition to SPMS as the disease progresses. SPMS will result in accumulating disability [105]. Approximately 10–15% of MS patients experience immediate disease progression, which defines the PPMS phenotype [105]. At the cellular level, this manifests as an early onset of neuro-axonal degeneration, which is the primary cause of long-term disability across all MS phenotypes [105,107,108]. Although the primary etiology of MS is still debated, there is general agreement that the disease is multifactorial, involving genetic predisposition and environmental triggers. With regard to the latter, it is accepted that viral infection can be an induction factor. For example, Epstein–Barr virus (EBV; a.k.a., HHV-4) and roseolovirus (HHV-6) infections are strongly implicated in MS. In terms of the latter, serological tests indicate that the HHV-6A subtype appears to be more strongly associated with MS than HHV-6B. However, since most clinical studies do not differentiate between HHV-6A and HHV-6B, excluding HHV-6B as an etiologic agent requires further study [39,109,110].

Early neuropathological studies provided evidence for direct HHV-6 involvement in MS by demonstrating viral DNA and antigen expression within demyelinating plaques isolated from MS brain tissue [108]. These findings support the presence of active or latent viral infection within CNS lesions rather than incidental peripheral exposure. Subsequent quantitative analyses reinforced this association, reporting that ~50% of individuals with MS showed evidence of infection with one or more human herpesviruses.

Importantly, exposure to EBV, HHV-6, and varicella-zoster virus (VZV) is associated with an increased likelihood of MS compared with control populations, suggesting that different herpesviruses play a role in MS susceptibility during polyviral infections [109].

Mechanistically, it is proposed that HHV-6 (i.e., HHV-6A) influences pathogenesis in MS through several complementary pathways. These mechanisms include modulation of T-cell responses, alteration of cytokine networks, and direct effects on CNS resident cells [66,110,111,112,113]. HHV-6A has been shown to readily infect astrocytes, oligodendrocytes, and microglia, yielding changes in inflammatory signaling and cellular homeostasis that may promote demyelination and neuroimmune dysregulation [66,111,112]. Oligodendrocyte dysfunction, a characteristic feature of MS, may be aggravated by virus-driven cell stress and inflammatory mediators, further impairing myelin maintenance and repair [112,113].

More recent clinical studies have strengthened the links between HHV-6 and MS. Patients with RRMS exhibit elevated antibody responses to reactivation-related antigens of both EBV and HHV-6, including antibodies targeting lytic phase viral proteins [106,107]. These heightened immune responses correlate with markers of systemic inflammation and with clinical disability, as assessed by standardized measures such as the Expanded Disability Status Scale (EDSS). This suggests that herpesvirus reactivation rather than simply seropositivity may be relevant to disease progression [106]. Together, data support a model in which HHV-6 acts as a factor in MS neuroinflammation and demyelination.

## 4. HHV-6 and Epileptogenesis

Evidence across acute, chronic, and degenerative neurological conditions supports a model in which roseolovirus neurotropism, cellular specificity, and immune modulation converge to alter CNS function. While disease manifestations may differ clinically, shared mechanisms, astrocyte dysfunction, neuroinflammation, excitatory–inhibitory imbalance, and viral latency offer a unifying framework for HHV-6-mediated neurological disease.

Indeed, the latest research implicates HHV-6 as a putative agent of chronic metabolic and neuroimmune dysfunction, with viral infection dynamics that overlap with pathways implicated in epilepsy and other neurological disorders.

Many of the afore-mentioned factors associated with roseolovirus–host dynamics, such as: differential cell tropism; cycles of viral latency and triggered virus reactivation; demyelination; lesions; scarring; neuronal network signal perturbations; and virus-driven immune system modulation—have a potential role in acute and chronic seizure disorders, including clinically defined epilepsy. This section provides an overview of what is known about roseolovirus infection and seizure disorders followed by a more focused review of HHV-6 infection and disruptions to neural systems of the mesial temporal lobe (MTL).

### 4.1. HHV-6 and Seizure Disorders

The association between HHV-6 infection and seizure disorders is documented in both pediatric and adult patient populations with febrile and chronic seizure phenotypes, respectively. A systematic review reported that in ~21% of children with febrile seizures as well as a subset of pediatric patients with more severe seizures (i.e., status epilepticus), HHV-6 was detected [3]. These reports support a role for HHV-6, particularly HHV-6B, in acute seizure susceptibility during infant and early childhood roseolovirus infection. The detection of HHV-6 in brain tissue from adults with epilepsy has generated ongoing debate regarding causality versus incidental presence. Studies using resected temporal lobe tissue reveal that while the prevalence of HHV-6B DNA did not differ significantly between temporal lobe epilepsy (TLE) patients and controls, viral loads were markedly higher in samples from TLE patients (*p* < 0.001), suggesting that viral burden rather than simple presence may be relevant [87]. These findings raise the possibility that HHV-6 may contribute to epileptogenesis or exacerbate epilepsy via sustained or reactivated infection. At the cellular level, herpesviruses including HHV-6 can induce neuronal injury and cell death, particularly in vulnerable or immunocompromised individuals.

Viral infection can alter neuronal signaling by affecting receptor expression, ligand availability, and ion channel function, thus disrupting synaptic homeostasis and lowering the threshold for seizure induction [9,22]. Collectively, such mechanisms offer a framework through which HHV-6 infection may cause seizure activity.

### 4.2. HHV-6 and Mesial Temporal Sclerosis

Mesial temporal sclerosis (MTS), a defining pathological feature of drug-resistant MTLE, has been extensively examined for a viral-based etiology. In a landmark study, HHV-6B DNA was detected in resected hippocampal tissue in 29 of 54 patients with MTS, suggesting a strong association between roseolovirus infection and a key marker of epileptogenic pathology [26]. Although causality is not firmly established, data suggest that HHV-6 contributes to epilepsy via inflammation and glial dysfunction mechanisms.

A subsequent meta-analysis supports an association between HHV-6B and epilepsy by showing significantly higher HHV-6B detection rates in MTLE patients compared to controls across multiple studies (OR = 9.42, 95% CI: 3.66–24.25, *p* < 0.00001) [114]. The same analysis found no significant association between HHV-6B infection and convulsion preceding MTLE, indicating that viral involvement may operate independently of initial seizure events. Given that HHV-6B preferentially infects astrocytes, oligodendrocytes, and microglia, viral persistence in cell populations may impair glial–neuronal signaling and promote characteristic structural remodeling characteristic observed in MTS [114].

Pediatric studies further highlight HHV-6 as an underrecognized factor in seizure disorders. In a cohort of children with febrile and epileptic seizures, HHV-6 was the most frequently identified pathogen, detected in approximately one-third of patients [115]. HHV-6 seropositive patients show elevated inflammatory markers like C-reactive protein (CRP) and procalcitonin, reflecting systemic inflammatory responses during infection.

### 4.3. HHV-6 and Temporal Lobe Epilepsy (TLE)

Data linking roseolovirus-induced inflammation to seizure susceptibility prompts consideration of HHV-6 infection in clinical evaluation of pediatric seizures [115].

Beyond acute seizure disorders, increasing evidence implicates HHV-6, particularly HHV-6B, in the longer-term pathogenesis of epilepsy, including TLE. Both viral DNA and antigens have been detected in hippocampal and amygdalar tissue from TLE patients, many of whom exhibit hippocampal sclerosis and a history of recurrent seizures [23,25,26,27]. Mechanistically, HHV-6 infection may contribute to epileptogenesis by inducing neuronal injury, disrupting synaptic circuitry, and activating pro-inflammatory signaling pathways such as NF-κB and IL-17A, which promote glial activation and neuronal hyperexcitability [23]. Latent HHV-6 infection within the CNS introduces the possibility that reactivation of roseolovirus serves as a chronic driver of seizure susceptibility. In adults with epilepsy, HHV-6 DNA was detected more frequently in patient cohorts than in controls, and active viral replication was observed exclusively in the epilepsy group [116]. In similar studies, elevated levels of pro-inflammatory cytokines, particularly TNF-α, IL-1b, and IL-10 also correlated with increased viral loads (see Table 5), suggesting that immune amplification upon viral reactivation contributes to seizure and disease progression [10].

Studies indicate alteration of mesial temporal lobe structures in HHV-6-associated epilepsy. HHV-6 can remain latent in the hippocampus and amygdala, regions critically involved in seizure initiation and propagation, and viral reactivation has been associated with MTLE, especially in patients with hippocampal sclerosis and infant and childhood febrile seizures [116]. A meta-analysis reported the detection of HHV-6 DNA in 19.6% of TLE patients versus 10.3% in controls (*p* < 0.05). This further supports a pathogenic role for roseoloviruses in epilepsy and epileptogenesis [15].

### 4.4. Mechanisms of Seizure Induction and Epileptogenesis

Seizures emerge from disruption of the finely regulated balance between neuronal excitation and inhibition [26]. Under physiological conditions, this balance is maintained through inhibitory interneurons, ion gradients, and tightly controlled cellular membrane potentials. Perturbations such as the dysfunction of sodium–potassium ATPase activity, elevated extracellular potassium, or impaired glial ion buffering can precipitate excessive neuronal firing. Genetic or acquired alterations in voltage-gated sodium channels may alter action potential thresholds, inducing seizure activity [117].

**Figure 2:**
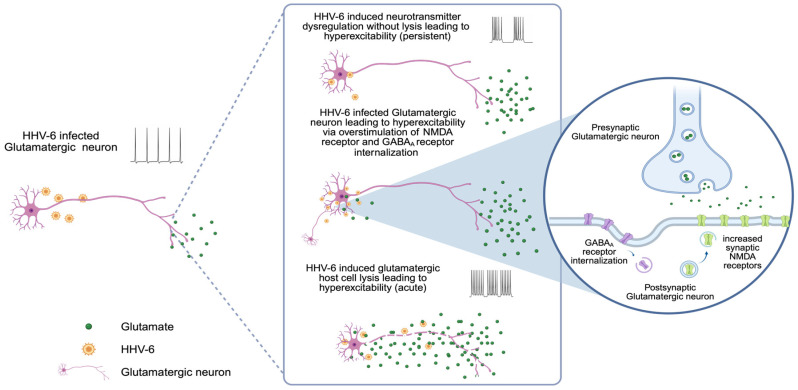
Scenarios of HHV-6-induced hyperexcitability during primary infection. Schematic depicts three scenarios by which HHV-6 infection of glutamatergic neurons can lead to hyperexcitation. Persistent infection (without gross cell lysis) can dysregulate glutamatergic regulation, resulting in chronic hyperexcitability (top). HHV-6 infection may increase glutamate release by inducing the upregulation of NMDA receptor expression while decreasing inhibitory signaling through GABA_A_ receptor internalization (middle). Alternatively, infection may cause gross host cell lysis with an acute release of glutamate and network hyperexcitation (bottom).

Synaptic transmission plays a central role in epileptogenesis; however, the respective contributions of excitatory versus inhibitory neurotransmitter pathways are decidedly context-dependent (Figure 2). While glutamate mediates excitation and GABA mediates inhibition, GABA signaling can paradoxically depolarize neurons under some conditions, such as early development or pathological states involving perturbed chloride gradients regulated by NKCC1 and related transporters [31]. Similarly, glutamatergic transmission may either promote excitation or recruit inhibitory circuits depending on receptor subtype expression or network architecture, complicating its net effect on seizure generation [31].

Beyond intrinsic excitability, seizure induction requires synchronization across the involved neuronal networks. Paroxysmal depolarization shifts (PDS), characterized by synchronized bursts of activity in pyramidal neurons, are a hallmark electrophysiological feature of seizure activity [32]. Synchronization emerges through multiple interacting mechanisms, including the dense glutamatergic connectivity among pyramidal neurons, electrical coupling via gap junctions, and the paradoxical synchronization mediated by inhibitory interneurons [39,40,43]. In TLE, experimental models exhibit a pronounced loss of neurons within limbic regions, particularly the hippocampus and entorhinal cortex. Vulnerable populations include dentate hilar neurons and layer III pyramidal neurons of entorhinal cortex, while certain GABAergic neurons show resistance to injury [118,119,120]. 

The hyperexcitability of layer II stellate cells in the entorhinal cortex generates excessive synchronous input to dentate granule cells, producing large-amplitude field potentials that resemble characteristic interictal EEG spikes observed clinically in epilepsy [121,122,123,124].

**Figure 3:**
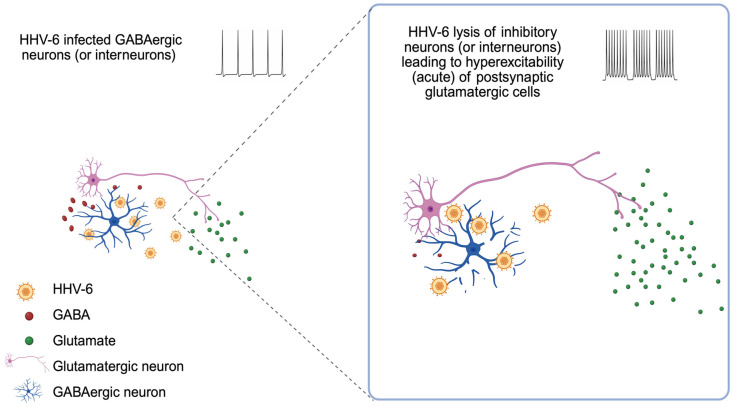
HHV-6-induced hyperexcitability via primary infection of GABAergic cells. A schematic depicting HHV-6 infection of a GABAergic neuron (or interneuron) providing inhibitory input to an excitatory glutamatergic neuron, resulting in GABAergic cell lysis or damage. The hypothesis of reduced inhibitory input from GABAergic neurons to postsynaptic glutamatergic neurons results in increased release of glutamate and hyperexcitability in the downstream network.

Therefore, the dormant interneuron hypothesis offers an additional framework for understanding epileptogenesis (Figure 3). Under this premise, a reduced excitatory drive or selective infection of GABAergic cells renders inhibitory inputs functionally inactive, thus diminishing inhibitory control and promoting network hyperexcitability [125]. This hypothesis is extended to extrahippocampal regions, including the entorhinal cortex, and highlights the distributed nature of epileptic networks [126]. Within this context, HHV-6 infection may amplify epileptogenic processes by targeting glia and neurons, promoting neuroinflammation, thereby destabilizing synaptic and network homeostasis. Therefore, virus-mediated effects likely interact with pre-existing vulnerabilities to drive the development and persistence of epileptiform activity.

## 5. Mechanisms of Seizures in Temporal Lobe Epilepsy (TLE)

Temporal lobe epilepsy (TLE) is a complex neurological disorder characterized by recurrent seizures arising from persistently hyperexcitable circuits in the temporal lobe. Mechanistically, seizure generation in TLE reflects the interaction between long-standing interictal network instability and acute triggers that convert latent vulnerability into overt ictal activity. In this section, we review the general mechanisms known to be involved in TLE and provide a mechanistic framework describing how roseolovirus infection-mediated cellular and molecular disturbances may intersect with established pathways of seizure generation in TLE.

### 5.1. General Mechanisms in TLE

In TLE, a dominant epileptogenic focus may be identifiable. However, many patients with TLE exhibit multifocal dysfunction with multiple overlapping cellular and molecular mechanisms contributing to seizure initiation and propagation, particularly in individuals with high seizure frequency [127]. Mechanistic heterogeneity helps explain the limited efficacy of anticonvulsant therapies in TLE, as targeting a single pathway often leaves parallel epileptogenic processes intact.

Seizures in TLE may arise from diverse etiologies, including genetic predisposition, developmental abnormalities, and acquired insults such as traumatic brain injury, viral infections, prolonged febrile seizures, and ischemia [128,129,130,131]. Among acquired insults, neurotropic viral infections including those by HHV-6A and HHV-6B are increasingly recognized as biologically plausible contributors to epileptogenesis. Viral exposure can initiate a cascade of events beyond acute inflammation, including astrocytic dysfunction, disruption of glutamatergic or cholinergic signaling, sustained neuroimmune activation, and long-term remodeling of neuronal and glial gene expression. Collectively, these mechanisms define a vulnerable neurobiological landscape in the temporal lobe and other regions of the CNS that may be exploited by persistent or reactivated HHV-6 infection.

### 5.2. Acquired Epileptogenesis in TLE

Many individuals with TLE experience an initial precipitating injury followed by a clinically silent interval and a delay in the emergence of spontaneous recurrent seizures. Although this interval has traditionally been described as a latent period, accumulating evidence suggests that epileptogenesis may represent a continuous, progressive process rather than a dormant phase. Pathological changes initiated by the initial insult may evolve over time and require additional biological stressors or “second hits” to reach the threshold for clinical seizure expression [132,133]. Neuropathological studies consistently reveal neuronal loss as a defining feature of TLE, indicating that selective vulnerability among neuronal populations plays a central role in shaping epileptogenic circuits [134].

Hippocampal principal neurons are particularly sensitive to injury, whereas certain inhibitory interneuron subtypes show greater resilience. In parallel, secondary processes like synaptic reorganization, glial activation, circuit rewiring, and molecular remodeling, progressively reinforce hyperexcitability. In terms of HHV-6 infection, such changes may be further amplified by sustained immune signaling and glial dysfunction, accelerating the transition from injury to chronic epilepsy.

### 5.3. Neuronal Network and Single-Cell Dynamics

Seizure initiation in TLE often arises in discrete neuronal microcircuits composed of excitatory pyramidal neurons and inhibitory interneurons. Disruption of the fine balance between excitation and inhibition is a core feature of epileptogenic networks. Impairment of GABAergic interneurons is particularly important; parvalbumin-positive interneurons, which normally exert fast and powerful inhibitory control, exhibit functional deficits in both human TLE tissue and experimental models. The loss of interneuronal inhibitory restraint correlates with increases in network synchrony and a higher predisposition for seizure activity [135,136].

Advanced imaging approaches reveal that seizure activity will frequently originate from localized microdomains within the hippocampus that exhibit highly synchronized signaling seconds before ictal onset [137]. Methods such as two-photon calcium imaging and optogenetics demonstrate that selective manipulation of small neuronal ensembles can either precipitate or suppress seizure activity [138,139]. These findings emphasize that seizure generation is often initiated at the microcircuit level rather than across entire brain regions.

Non-neuronal cells contribute critically to microcircuit stability. Astrocytes regulate extracellular potassium levels, neurotransmitter availability, and metabolic coupling, while microglia influence synaptic signals via cytokine release and reactive intermediates. Dysregulation of glial function can amplify neuronal hyperexcitability and promote focal seizure initiation. In terms of HHV-6 infection, which may preferentially target glial cell populations, perturbing these regulatory processes may further destabilize epileptogenic microcircuits [140,141].

### 5.4. Individual Neuron Epileptiform Firing Patterns

At the cellular level, epileptiform activity is frequently characterized by paroxysmal depolarization shifts (PDS), consisting of prolonged depolarizations with superimposed bursts of action potentials (AP) followed by pronounced afterhyperpolarization. These electrophysiological events represent fundamental cellular correlates of ictal discharges and are commonly recognized as key markers of epileptogenic neurons [142]. Isolated pyramidal neurons in hippocampal and cortical slice preparations will commonly display spontaneous epileptiform discharges under hyperexcitable conditions when induced by agents such as 4-aminopyridine or kainic acid [143,144]. Intrinsic alterations in ion channel dynamics contribute substantially to cell firing abnormalities. An increased persistence in sodium currents, reduced potassium conductance, and the presence of ion channel variants including the KCNQ2/3 and SCN8A genes have been associated with inherited and acquired epilepsy [145,146].

Single-cell transcriptomic analyses further reveal molecular signatures of epileptic neurons, including the upregulation of excitatory signaling pathways and suppression of inhibitory programs [147]. High-density microelectrode recordings in animal models and freely moving systems demonstrate close temporal coupling between aberrant single-cell firing, pathological sharp wave–ripple activity, and behavioral seizures [148,149,150]. These intrinsic neuronal changes provide a cellular substrate upon which neuroinflammation or metabolic stress may act to exacerbate epileptiform activity during the course of a viral infection or other insult.

### 5.5. The Role of Glutamate in Seizures and Epileptogenesis

Glutamatergic neurotransmission plays a central role in seizure initiation as well as seizure-induced neuronal injury. During prolonged seizures and status epilepticus (SE), excessive acetylcholine release can facilitate recruitment of glutamatergic neural circuits, particularly in toxin-induced or viral encephalitic conditions [151]. Elevated extracellular glutamate overstimulates NMDA receptors, triggering sustained depolarization, calcium influx, and excitotoxic damage [152].

Extended seizure activity alters receptor trafficking, leading to internalization of GABAA receptors and increased synaptic incorporation of NMDA receptors. Receptor redistribution shifts the neural network balance toward excitation and contributes to pharmaco-resistance during SE [153]. These dynamics explain why GABAergic therapies alone are often insufficient in refractory seizures and why combined treatment with NMDA receptor antagonists improves seizure control [154]. In terms of HHV-6 infection, impaired glutamate clearance by infected astrocytes may amplify excitotoxic cascades.

### 5.6. Astrocyte Dysfunction

Astrocytes are essential for maintaining glutamate homeostasis, potassium buffering, metabolic support, and synaptic stability in the CNS. Disruption of astrocytic function is a prominent feature of TLE pathology. Astrocytes infected with HHV-6B were identified in resected brain tissue from patients with epilepsy of unknown etiology [26]. Infected cells exhibited morphological abnormalities indicative of functional impairment [26].

Viral infection can also alter astrocytic glutamate transporter expression particularly EAAT2 (a.k.a., GLT-1 in rodents) leading to elevated extracellular glutamate levels and secondary hippocampal injury, thereby contributing to mesial temporal lobe epilepsy (MTLE) [28,155]. Astrocytic dysfunction in TLE extends to metabolic pathways, including reduced glutamine synthetase, glutaminase, and glutamate dehydrogenase activity, thereby disrupting the glutamate–glutamine cycle [28].

**Figure 4:**
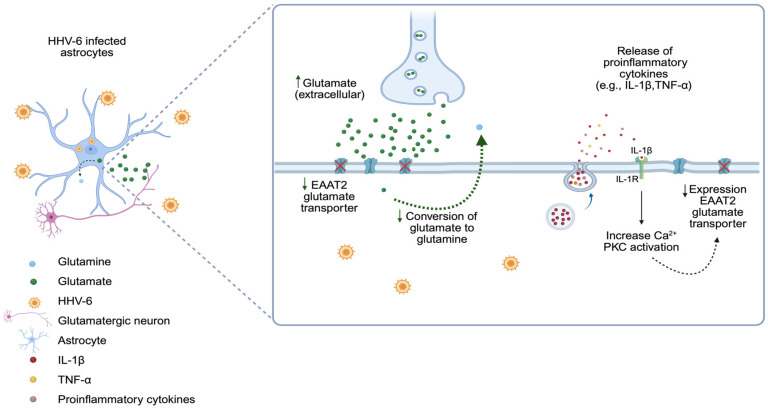
HHV-6-induced hyperexcitability via primary infection of astrocytes. Schematic depicts HHV-6 infection of an astrocyte, which impairs glutamate homeostasis at the synapse, leading to reduced EAAT2-mediated glutamate uptake via activation of pro-inflammatory cytokines and chemokines (e.g., IL-1β, TNF-α). EAAT2 dysregulation results in increased extracellular glutamate and network hyperexcitation. (Arrows indicate up- and down-regulation, respectively).

Disturbances in astrocyte function (Figure 4) may occur alongside characteristic TLE neuronal loss in the hippocampus and entorhinal cortex, where dentate hilar neurons and entorhinal layer III pyramidal cells exhibit marked vulnerability [118,119].

Despite this extensive neuronal degeneration, certain interneuron populations are relatively preserved [120], generating an imbalance that favors hyperexcitability. Indeed, hyperexcitability of entorhinal layer II stellate cells further drives excessive excitatory inputs to the dentate granule cells, producing field excitatory postsynaptic potentials resembling interictal spikes [121,122,123,124]. The dormant interneuron hypothesis proposes that reduced excitatory drive results in functionally inactive inhibitory interneurons, diminishing inhibitory tone and promoting seizure propagation [125,126].

### 5.7. Regulation of Seizure Activity: Dopamine, Norepinephrine, and Serotonin

**Figure 5:**
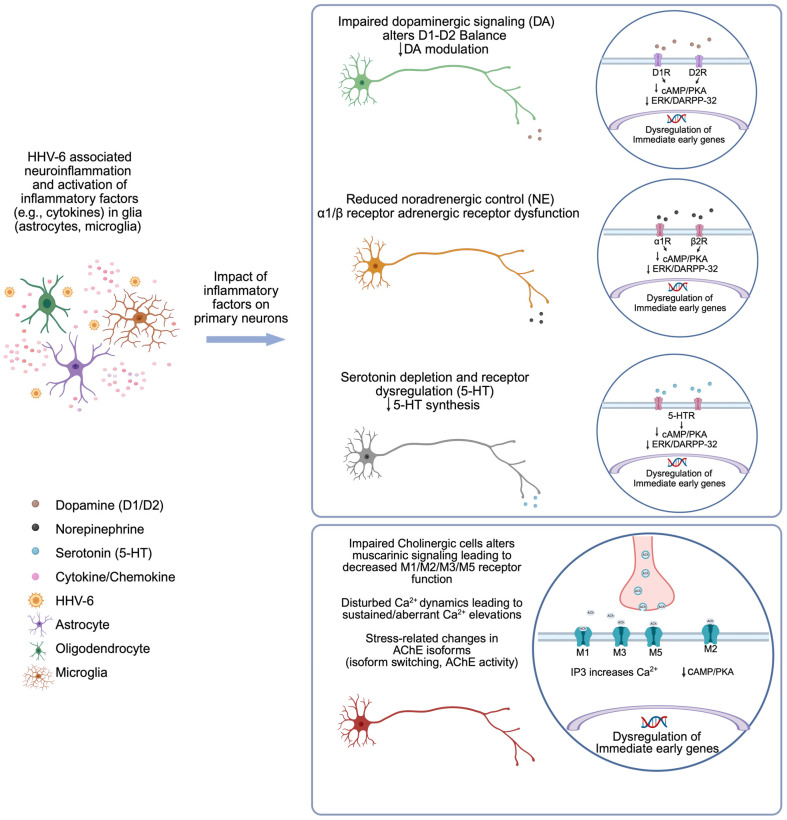
HHV-6 infection, inflammation, neuromodulation, and cholinergic control. Infection with HHV-6 can result in neuroinflammation and alterations to neuromodulation and cholinergic control in limbic systems. This schematic depicts a variety of mechanisms by which HHV-6 infection may contribute to seizure susceptibility by disrupting neuromodulatory systems that normally restrain limbic excitability. Activation of inflammatory pathways and release of pro-inflammatory cytokines and chemokines can dysregulate dopaminergic (DA), noradrenergic (NA), and serotonergic (5-HT) signaling, thereby altering downstream intracellular pathways and immediate early gene responses. In parallel, inflammation may indirectly disrupt cholinergic regulation of entorhinal–hippocampal circuits through alterations in muscarinic signaling, calcium dynamics, and stress-related changes in levels of acetylcholinesterase (AchE) isoforms. (Down arrows represent down-regulation).

Neuromodulatory systems add another layer of regulation to epileptogenic networks by influencing intracellular signaling pathways that ultimately shape neuron excitability. Dopamine (DA), norepinephrine (NE), and serotonin (5-HT) modulate seizure events via receptor-dependent mechanisms (Figure 5) involving cAMP/PKA signaling, ERK activation, and DARPP-32 phosphorylation [156,157].

DA exerts bidirectional effects on seizure regulation. Activation of D1-like receptors enhances excitability via adenylate cyclase stimulation, increased cAMP production, and induction of immediate early genes such as c-fos and zif268 [158,159,160].

In contrast, D2-like receptor activation generally suppresses excitability by reducing cAMP signaling and modifying DARPP-32 phosphorylation [161,162]. Dopaminergic signaling also activates ERK pathways. ERK pathways are rapidly engaged during seizure and will contribute to activity-dependent transcriptional responses [160].

NE originating from the locus coeruleus (LC) can modulate limbic excitability and seizure-associated gene expression. Lesions of the locus coeruleus reduce seizure-induced Fos expression, underscoring a modulatory role of noradrenergic input in shaping seizure dynamics [163]. 5-HT similarly influences susceptibility to seizure. A depletion of 5-HT predisposes epileptogenic circuits to seizure by lowering thresholds, whereas enhanced serotonergic tone suppresses epileptiform activity [164]. Serotonergic receptors 5-HT1A and 5-HT2C regulate excitability via G protein-coupled receptor pathways and influence immediate early gene expression linked to synaptic plasticity [62,165,166,167,168]. NE and 5-HT neuromodulatory systems converge on shared intracellular pathways that govern both acute seizure expression and long-term network remodeling [169,170,171].

### 5.8. Cholinergic Dysfunction in Temporal Lobe Epilepsy

Cholinergic signaling plays a key role in regulating entorhinal–hippocampal circuit excitability. The entorhinal cortex integrates multisensory input and projects efferents to hippocampal subfields through anatomically organized pathways, with layer III neurons influencing CA1, CA3, and subicular pyramidal cells as well as feed-forward inhibitory networks [172,173]. Acetylcholine (ACh) availability determines functional network state. Elevated ACh promotes theta and gamma oscillations that support synaptic plasticity and information processing and modulates interneuron excitability and dendritic integration [174,175,176,177,178]. Reduced ACh has been reported in several epilepsy models and may reflect altered acetylcholinesterase expression, receptor dysfunction, or damage to cholinergic projections [175,179]. Viral encephalitis, including that caused by herpesvirus infections, is associated with reductions in cholinergic markers, particularly in severe cases [180].

Activation of muscarinic acetylcholine receptors can elevate intracellular calcium, triggering transcriptional programs that influence cellular metabolism, excitability, and survival [181,182]. Following seizure activity, Ca^2+^-dependent mechanisms attenuate the expression of choline acetyltransferase (ChAT) and vesicular ACh transporter proteins, while acetylcholinesterase (AChE) expression can increase through alternative splicing [183]. Although interpreted as compensatory, altered receptor distribution and signaling efficiency may paradoxically enhance neuronal excitability [179]. For select neurotropic viruses (e.g., Bornavirus), cholinergic pathways appear particularly vulnerable to insult and injury [184], supporting the plausibility that HHV-6-mediated glial or inflammatory injury may indirectly disrupt cholinergic regulation in TLE.

### 5.9. Neuroinflammatory Pathways in Epilepsy

Neuroinflammation plays a central role in the initiation and amplification of epileptic activity [9,141]. Inflammatory responses may arise from infection, autoimmunity, toxic exposure, or neuronal hyperactivity via a process called neurogenic neuroinflammation [185]. Several pathogens, including neurocysticercosis, toxoplasmosis, fungal agents, and prions, have been implicated in status epilepticus and subsequent epilepsy development [186,187]. Multiple viruses are also known to infect the CNS, causing neural dysfunction. Acute infection by viruses like Japanese Encephalitis Virus (JEV) and Borna Disease Virus (BDV) induces inflammatory responses leading to neuro-encephalitis, which, in turn, can result in seizure [180,184]. Primary infection by HHV-1 (herpes simplex virus, HSV-1), HHV-4 (Epstein–Barr virus, EBV), or HHV-5 (cytomegalovirus, CMV) as well as HHV-6A and HHV-6B can also induce neuro-encephalitis, facilitating acute seizure activity [15,46]. However, the roseoloviruses (i.e., HHV-6A and HHV-6B) are unique in that they exhibit notably high seroprevalence in human populations and exact nervous system dysfunction via chronic infection that may transform from latency to reactivation, thus inducing neuronal injury, glial dysfunction, circuit remodeling, and long-term alteration in neuron signaling [11,12,13,14,15,20,21,31,32,33].

Thus, the importance of roseolovirus-induced epileptogenesis comes not from a unique biology in terms of inducing neuro-inflammatory responses but instead from their high seroprevalence and capability to trigger specific seizure-promoting pathways during a reactivation event after long periods of latency.

Between the initial insult and the emergence of spontaneous seizures, brain tissue undergoes extensive remodeling characterized by blood–brain barrier (BBB) disruption, synaptic reorganization, neuronal loss, gliosis, and epigenetic modifications [22]. In the context of HHV-6 infection, persistent immune activation and glial infection may sustain inflammatory signaling during this interval, lowering thresholds to seizure and inducing long-term epileptogenic remodeling (see Table 7).

## 6. HHV-6-Induced Mechanisms of Epileptogenesis

HHV-6 has emerged as a biologically plausible contributor to epileptogenesis through its capacity to establish persistent infection in the CNS and to perturb multiple cellular systems, which are critical for neuronal signaling and neural network stability. Rather than acting through a single pathway, HHV-6 may promote seizure susceptibility by additive or synergistic mechanisms, including neurotransmitter imbalance, astrocyte dysfunction, neuroimmune system modulation, and altered neuronal excitability. Indeed, there are several hypotheses regarding roseolovirus-mediated epileptogenesis (Table 7). These mechanisms are relevant to temporal lobe epilepsy (TLE), where viral DNA and protein have been detected in hippocampus and amygdala and where glial pathology and neuroinflammation notably occur. In this section, we relate the impacts of roseolovirus infection in the CNS to working hypotheses regarding HHV-6-mediated epileptogenesis.

### 6.1. Astrocytic Dysfunction, Gliosis, and Neuronal Loss

Astrocytes are key regulators of extracellular glutamate, potassium homeostasis, and metabolic coupling between neurons and glia [155]. Evidence from resected epileptogenic tissue shows that HHV-6B preferentially infects astrocytes and results in morphological abnormalities as well as functional impairment [26,29]. Roseolovirus-infected astrocytes exhibit dysregulation of the excitatory amino acid transporter EAAT2, which leads to a reduced capacity to clear glutamate and elevated extracellular glutamate levels [28,155]. Excess extracellular glutamate may hyperexcite glutamatergic neurons leading to seizure. In parallel, the loss of astroglial glutamine synthetase and alterations in glutaminase and glutamate dehydrogenase disrupt the glutamate–glutamine cycle, further compromising neurotransmitter homeostasis [28]. Astrocytic disturbances create a permissive environment for sustained excitation and excitotoxic injury, particularly in the hippocampus. During prolonged seizure activity or status epilepticus, NMDA receptor hyperactivation coincides with internalization of GABA_A_ receptors, amplifying the excitatory–inhibitory imbalance and promoting chronic seizure susceptibility.

Consistent with this model, HHV-6 viral load is highest in hippocampal regions, which are the areas of the mesial temporal lobe most vulnerable to neuronal loss in MTLE. Astrocyte infection with HHV-6 is closely associated with reactive gliosis and neuronal degeneration. Increased expression of glial fibrillary acidic protein (GFAP) and CCL-2 has been observed in roseolovirus-positive MTLE tissue, correlating with viral burden and neuronal loss [27,188,189,190]. CCL-2-mediated recruitment of monocytes and macrophages may facilitate the trafficking of infected cells into limbic structures, thus sustaining viral persistence and chronic inflammation in epileptogenic networks [191].

### 6.2. Neurotransmitter Imbalance and Circuit Hyperexcitability

HHV-6-related astrocytic pathology has downstream consequences for excitatory and inhibitory neurotransmission. Impaired glutamate uptake yields prolonged synaptic glutamate signaling and NMDA receptor overactivation, while disruption of glutamine supplies reduces GABA synthesis, weakening inhibitory tone [208,209]. This dual effect shifts network dynamics toward hyperexcitability, particularly within hippocampal and entorhinal circuits, which are central to seizure generation in TLE.

Although direct infection of GABAergic neurons by HHV-6 appears limited [126], indirect effects mediated by astrocyte dysfunction and metabolic disruption are sufficient to compromise inhibitory control. Reduced efficacy of interneuron-mediated inhibition aligns with observations from experimental models, in which selective vulnerability of excitatory neurons, combined with relative preservation of certain interneuron subtypes, results in aberrant synchronization and seizure propagation [126].

Cholinergic signaling may represent an additional, indirect pathway through which HHV-6 influences seizure susceptibility. While direct evidence demonstrating HHV-6 in cholinergic neurons is limited, data from other neurotropic viruses, including HSV-1 and Borna disease virus (BDV), suggest that viral infection can disrupt cholinergic innervation and cholinergic function, thus altering acetylcholine availability and muscarinic receptor signaling [26,29,180,184].

Altered calcium dynamics downstream of muscarinic receptor activation may also enhance excitability in entorhinal and hippocampal neurons, which are cells particularly sensitive to Ca^2+^-dependent plasticity and excitotoxic stress [194]. Additionally, changes in acetylcholinesterase (AChE) splicing during infection may favor stress-induced AChE isoforms, thus reducing synaptic acetylcholine and lowering seizure thresholds [183].

### 6.3. Neuromodulatory System Disruption

Beyond classical neurotransmitters, HHV-6 infection can impact neuromodulatory systems that exert control over seizure susceptibility. Dopaminergic, noradrenergic, and serotonergic pathways modulate neuronal excitability via shared intracellular signaling cascades, including cAMP/PKA, ERK, and immediate early genes. Neuroinflammation from HHV-6 infection can impair these systems, weakening endogenous anticonvulsant mechanisms [67,192,193].

Roseolovirus-mediated disruption of dopaminergic signaling may alter the balance between D1- versus D2-mediated effects on excitability, while impaired norepinephrine release from the locus coeruleus reduces regulatory control over limbic circuits [157,163]. Likewise, serotonergic depletion or receptor dysregulation is associated with low seizure threshold and enhanced network synchrony [164]. These neuromodulatory disturbances amplify impacts of local circuit pathologies and contribute to seizure propagation.

### 6.4. Neuroimmune Activation and Inflammatory Signaling

Immune activation represents a key axis linking HHV-6 infection to epileptogenesis. HHV-6 engages host immune pathways through its interaction with CD46 and CD134 cell surface receptors, which are expressed on neural and immune cells, including neural stem cells [59,60,67,195]. Viral DNA can also activate pattern-recognition receptors such as Toll-like receptor 9 (TLR9), initiating MyD88-dependent signaling cascades that culminate in NF-κB activation and transcription of proinflammatory cytokines [102,196,197,198,199,200,201,202,203].

HHV-6-infected astrocytes and oligodendrocytes increase levels of IL-1β, TNF-α, IFN-α, and chemokines including CCL-2, CCL-5, and CXCL-2 [66,86]. These cytokines directly impair glutamate transporter function, exacerbate excitotoxicity, and promote neuronal injury [204]. Although anti-inflammatory counter-regulation via IL-10 has been observed following HHV-6A infection in vitro, this response is insignificant or absent in HHV-6B infection, allowing sustained inflammation to persist [195,205]. NF-κB signaling is implicated in HHV-6-associated MTLE, linking viral-induced neuroinflammation to hippocampal sclerosis and chronic seizure susceptibility [65,206]. Elevated IL-8 levels in cerebrospinal fluid and saliva from seizure patients with roseolovirus infection further support a cytokine-driven contribution to neuronal dysfunction [82,207].

In addition to direct impacts on glutamate transport, inflammatory signaling may lower seizure threshold by connecting immune activation to activity-dependent neural circuit alteration. Proinflammatory mediators such IL-1β can impair glial glutamate buffering, leading to increased excitatory drive. Additionally, prolonged glial activation can create a local inflammatory environment, which in turn may be exacerbated by seizure activity [141,185,191,204]. In this scenario, HHV-6-associated neuroinflammation is more likely to work as a feed-forward mechanism in which cytokine release, excitotoxicity, and recurrent neuronal firing all magnify one another.

Over time, this may induce rearrangement in synapses, neuronal damage, and long-term epileptogenic remodeling, raising the risk of seizure [22]. HHV-6B found in MTLE tissues is linked to increased NF-κB signaling, suggesting a relationship between inflammation and a hyperactive limbic environment [65].

### 6.5. Electrophysiological Consequences of Viral Infection

Roseolovirus infections can fundamentally disrupt electrophysiological properties, reshaping neural network dynamics long before overt cell death occurs. Studies of other neurotropic viruses show that infection can initially induce hyperexcitability, followed by progressive silencing or dysregulated firing activity as intracellular signaling and calcium homeostasis are disrupted [63,210,211,212]. HHV-6 follows a similar trajectory, interfering with calcium signaling, synaptic transmission, and neurotransmitter release [2,29,101].

These electrophysiological alterations align with observed features of epileptogenic tissue, including abnormal synchronization in neural network firing, impaired inhibitory restraint, and susceptibility to paroxysmal depolarization shifts. Persistent roseolovirus infection in limbic circuits may therefore act as a chronic modifier of network excitability, lowering seizure threshold. This, in turn, may facilitate transitions from injury to epilepsy.

Although direct electrophysiological evidence demonstrating that HHV-6-infected nerve cells are sufficient for epileptogenesis, the data strongly suggest that roseolovirus infections lower seizure threshold, which is a mechanism for network hyperexcitability and a precursor to epileptic activity. Indeed, reducing the level of depolarization required for neuronal firing is demonstrated in several in vitro models.

For example, as described above (see Figure 4), HHV-6-infected astrocytes will down-regulate EAAT2 and impair glutamate clearance [28,29] resulting in hyperactivation of NMDA receptor activity concurrent with GABA_A_ receptor internalization [153,155]. This shifts the excitatory versus inhibitory balance in favor of postsynaptic neuron firing, which in turn can induce epileptiform network responses. This example alone suggests that roseolovirus infection can alter network activity leading to seizure.

Unlike transient cytokine surges observed in acute viral infections that resolve once the pathogen is cleared, persistent viral infections, like those occurring with HHV-6, may inducing long-term remodeling of susceptible neuronal networks subject to latency and reactivation cycles. This idea is supported by observations that sustained NMDA receptor activation induces changes in transcription and resets the baseline excitability of neurons as opposed to just a transient perturbation [147,160]. Moreover, infection of astrocytes can induce gliosis, which can chronically impair glutamate and potassium homeostasis, which reinforces the idea of virus-induced network remodeling and epileptogenesis [188,189,190].

## 7. Experimental Approaches to Investigate HHV-6 in Epileptogenesis

Understanding how HHV-6 contributes to epileptogenesis requires experimental models capable of capturing the interactions between neurotransmitter imbalance, virus infection, glial dysfunction, neuroimmune activation, neural network signaling changes, and virocell-omics profiles. Clinical and molecular evidence indicates that HHV-6 infects astrocytes, oligodendrocytes, and neurons, but the causal pathways linking infection to seizure onset remain under-defined. Targeted studies using human cell systems, brain organoids, animal models, and electrophysiologic methods are needed to address specific hypotheses regarding mechanisms by which HHV-6 induces or exacerbates MTLE. In this section, we discuss several quantitative strategies, culture systems, immunohistochemical techniques, electrophysiological methods, and in vivo approaches used to study impacts of HHV-6 infection on nerve cells to elucidate the neurochemical and neurophysiological substrates underlying seizure induction.

### 7.1. Virus Titers for MOI Experimental Infections

For experimental studies investigating mechanisms by which HHV-6 may contribute to epileptogenesis, calibration of viral inocula and considering the clinically relevant viral doses are imperative. Quantitative Polymerase Chain Reaction (qPCR) using primers that are specific for each HHV-6 subtype provides a sensitive approach for: estimating viral load; distinguishing HHV-6A from HHV-6B in biological specimens; and determining MOI for experimental infections [53,213]. Measuring HHV-6 DNA levels from patient samples, including serum and cerebrospinal fluid, can help define clinically relevant ranges of viral dose associated with neurologic disease [207]. In addition to qPCR-based viral gene copy number, viral titers can alternatively be measured by a 50% tissue culture infectious dose (TCID50). TCID50 provides a measure of infectious titer by calculating the quantity of replication-competent virus that is needed to infect 50% of cultured cells. Unlike qPCR, which counts viral DNA copy number, TCID relies on quantification of an HHV-6 infection marker. For in vivo studies, animal models of roseolovirus pathogenesis provide an important framework for relating experimental inocula in vitro to biologically meaningful infection outcomes. Human CD46-expressing transgenic mice can be used since CD46 functions as an HHV-6A cellular receptor as well as a secondary receptor for HHV-6B attachment and entry. These transgenic mice facilitate the study of roseolovirus neuroinvasion, persistence in the brain, and related inflammatory responses, making them an important platform for understanding the mechanisms by which HHV-6A may contribute to epilepsy [59,102]. However, infectious dose measurements such as ID50 should be interpreted within the context of the specific animal model and assay used [214].

### 7.2. Model Systems for Studying HHV-6-Associated Epileptogenesis and Data Integration

HHV-6 exhibits tropism for astrocytes and neurons. It is associated with gliosis and neuronal loss in brain tissue [67]. Differentiated human neural stem cells, immortalized cell lines, primary cultures from humanized mice, human brain tissue, organotypic slices, and three-dimensional human brain organoids (or hydrogels) containing mature neurons, astrocytes, and microglia represent physiologically relevant platforms for detailed studies of neurotropic virus infection. Such systems permit evaluation of reactive gliosis, cytokine induction, stress responses, and progressive increase in neuronal vulnerability over time.

In addition to glial and inflammatory responses, some of these platforms provide a means to test whether HHV-6 affects neuromodulatory systems that shape hippocampal excitability, particularly cholinergic signaling. Although direct evidence linking HHV-6 to cholinergic dysfunction is limited, viral encephalitis caused by herpes simplex virus (HSV) and Bornavirus demonstrates profound impacts on cholinergic signaling [180,184]. Future experimental investigation could involve infecting cholinergic neurons derived from human stem cells with HHV-6 and measuring changes in choline acetyltransferase (ChAT), acetycholine esterase (AChE), vesicular ACh transport, synaptic release, and muscarinic receptor sensitivity.

Studies in primary cell cultures or brain slices from hCD46+ transgenic mice targeting forebrain regions or in human neural stem cell lines that are differentiated to cholinergic end points subjected to HHV-6 infection may clarify whether roseoloviruses perturb ACh availability, induce epileptiform activity resulting in altered seizure thresholds, or disrupt hippocampal-dependent circuit functions related to MTLE [102,214].

Disruption of GABAergic and glutamatergic balance represents another potential mechanism of HHV-6-mediated epileptogenesis. HHV-6 reduces EAAT2 expression, thus impairing glutamate uptake in astrocytes. This elevates extracellular glutamate and drives excitotoxicity [28,29]. HHV-6 DNA and viral protein have been identified in hippocampal neurons from MTLE patients, suggesting direct viral impacts on neuronal metabolism and synaptic signaling [29]. 

Experimental systems such as mixed neuronal cultures or brain organoids infected with HHV-6 would allow assessment of changes in glutamate and GABA receptors, vesicular transporters, synaptic proteins, and inhibitory vs. excitatory synaptic strength. Electrophysiological recordings from mixed cell cultures prepared by differentiating and infecting GABAergic and glutamatergic iPSCs could elucidate and quantify changes in synaptic transmission, paroxysmal bursting, and network excitability [126]. Prolonged calcium imaging or multi-electrode array (MEA) recordings combined with microscopy analyses could reveal changes in astrocyte–neuron and neuron–neuron physical interactions, glutamate homeostasis, alterations in neuron-neuron signaling, and network hyperexcitability. Using patient-derived brain cells from HHV-6-positive MTLE cases could identify individual susceptibility variables influencing viral effects [67].

### 7.3. Comparative Virology

Another hypothesis is that HHV-6 contributes to neurologic disorders not only as the principal etiologic agent, but that roseoloviruses may also induce neurological disorders by interacting with other viruses, thereby exacerbating dysfunction induced by another pathogen. Simple reactivation of HHV-6 as a consequence of inflammation due to another primary cause, may push neural systems beyond a minimum threshold for disease presentation or contribute to the progression of a disease. This concept is supported by research in other systems, which demonstrates that polyviral interactions can exacerbate neurological dysfunction. For example, HSV-1 infection has been linked with increased intracellular amyloid-beta 1–40 (Aβ1–40) and amyloid-beta 1–42 (Aβ1–42) accumulation in neuronal and glial cells, as well as Aβ1–42 deposits in the mouse brain [215].

HHV-6A infection of microglial cells is linked to increased Aβ expression, microglial activation, and tau-related changes [2]. Both clinical and experimental data increasingly suggest an overlap between HSV-1 and HHV-6 in Alzheimer’s disease-related studies.

Overlapping viral infections can result in: an increased mixed herpesvirus load; higher frequency of reactivation or repeat infection; or additive or synergistic effects on neuroinflammation and proteinopathy [216]. Other polyviral infection scenarios offer an even greater potential for neuropathologies, as neurologic symptoms may become more severe or more frequent than what would present in a single-virus infection. For example, HIV-1/HTLV-1 co-infection has been connected to other neurological disorders including myelopathy and peripheral neuropathy. SARS-CoV-2 (COVID-19) infection has been linked to herpesvirus reactivation in patients with severe neurologic symptoms [217,218]. Although these examples do not suggest that HHV-6 coinfection causes epileptogenesis, they support the idea that viral coinfection or reactivation exacerbates CNS inflammation, decreases the threshold for neuronal damage, and accelerates neural disease progression.

## 8. Conclusions

Since the initial description of HHV-6 (a.k.a., human B-lymphotropic virus, HBLV) and the later reclassification of HHV-6 into two distinct viruses, HHV-6A and HHV-6B, it has become evident that these roseoloviruses with near-universal presence in the human population (>90% seroprevalence) may be involved as etiologic agents or complicating factors in a suite of common neurological disorders, including epilepsy. Indeed, recent data support a multifactorial model whereby HHV-6 infection contributes to epilepsy and epileptogenesis. This may occur as a result of differential host tropism between HHV-6A and HHV-6B and neurotransmitter dysregulation, via glial pathology, or virus-induced immune-mediated injury. Rather than acting through a single dominant mechanism, HHV-6B may create a permissive environment for seizure development by reshaping neuronal circuits and inflammatory signaling over time, whereas HHV-6A may result in more acute effects, directly inducing cell death and lesions.

In this review, we have summarized some of the current evidence linking HHV-6 to general neurophysiological and neurochemical dysfunction, thereafter associating each form of dysfunction to known diseases. We then focused on how roseolovirus-induced neurological dysfunction may contribute to the epileptogenesis and acquired epilepsy. Finally, we define several gaps in the literature that prevent a holistic understanding of the specific mechanisms by which HHV-6 infection leads to epilepsy and suggest a suite of experimental strategies that may serve to resolve unanswered questions and address hypotheses regarding roseolovirus-induced epilepsy, with a particular emphasis on mechanistic pathways underlying MTLE and viral-induced epileptogenesis.

A substantial body of clinical, molecular, and neuropathological evidence supports an association between HHV-6B and seizure disorders, particularly TLE. HHV-6B DNA and proteins have been identified in surgically resected hippocampal and amygdala tissue from patients with MTLE, often at significantly higher viral loads than in control samples [4,23,87,116]. Since HHV-6B can infect multiple cell types relevant to epilepsy, including astrocytes, oligodendrocytes, and neurons, and its presence in these cells has been linked to gliosis, inflammation, metabolic impairment, and selective neuronal vulnerability within epileptogenic circuits [25,27,67,71,86,188,189,190], the etiologic potential of HHV-6B in epileptogenesis is clearer than for HHV-6A.

Despite significant evidence that HHV-6 is associated with seizure disorders, current evidence suggests that no single pathway fully accounts for the relationship between HHV-6 and epilepsy. Instead, multiple mechanisms appear to interact, and their relative contributions likely vary among individuals depending on genetic background, immune status, prior neurological injury, and the specific viral species involved [10,24,114].

This mechanistic diversity explains why HHV-6B is strongly associated with epilepsy in some studies, while in others, HHV-6 appears restricted to subgroups or presents with variable pathological signatures. With this review, we hope to facilitate future work towards a more holistic understanding of how HHV-6A versus HHV-6B may act independently, in concert with one another, or in concert with other neurotropic viruses in epilepsy and other neurological diseases.

## Figures and Tables

**Table 1:** CNS cell types reported to harbor HHV-6A and/or HHV-6B: Glia.

Specific Cell Type	HHV-6 Species Reported	Study System	Key Finding [Reference]
Astrocytes	HHV-6 (unspecified)	Human MTLE surgical resections	HHV-6 detected in cells morphologically consistent with astrocytes in MTLE tissue [25]
	HHV-6B	Human MTLE/MTS tissue and derived astrocyte cultures	HHV-6B associated with MTLE; includes hippocampal temporal lobe astrocyte culture evidence [62,63]
	HHV-6B	Human MTLE hippocampal tissue	HHV-6B antigen in MTLE was reported mainly in cells morphologically resembling astrocytes and microglia [64,65]
	HHV-6A	Human adult astrocyte cultures	HHV-6A altered the cytokine-response network of adult human astrocytes under inflammatory stimulation [66]
	HHV-6A and HHV-6B	Differentiated human neural stem cell-derived cultures	Both viruses infected glia as well as neuronal cell types; glial infection included astrocyte-lineage cells [67]
	HHV-6	Human olfactory pathway encephalopathy tissue	HHV-6-positive astrocytes were identified, and conspicuously found in white matter [68]
Oligodendrocytes	HHV-6	Brains of children with AIDS encephalopathy	HHV-6 DNA was found primarily in oligodendrocytes of white matter, but with less frequency in astrocytes, macrophages, microglia, and neurons [69]
	HHV-6	Human olfactory pathway encephalopathy tissue	Oligodendrocytes were among the most frequently HHV-6-positive CNS cell types; the study concluded they were among the most severely affected targets [68]
	HHV-6B	Human infant autopsy with primary encephalitis	HHV-6B-infected oligodendrocytes with some neuronal and vascular endothelial cell infection [70]
	HHV-6	Cultured human neural cells	Direct infection effects were studied in cultured human oligodendrocytes [28]
Microglia	HHV-6	Brains of children with AIDS encephalopathy	HHV-6-positive microglia were detected less frequently than oligodendrocytes [69]
	HHV-6	Cultured human neural cells	Direct infection effects were studied in cultured human microglia [28]
	HHV-6B	Human MTLE hippocampal tissue	HHV-6B immunoreactivity was observed in cells morphologically resembling astrocytes and microglia [64,70]
	HHV-6A	Human microglia	HHV-6A infection induced Aβ-related and activation associated changes in microglia [2]
	HHV-6A and HHV-6B	Cerebellar cortex in mood-disorder brains	Occasional HHV-6-positive microglia were reported near infected Purkinje cells [71]

**Table 2:** CNS cell types reported to harbor HHV-6A and/or HHV-6B: Neurons and Others.

Specific Cell Type	HHV-6 Species Reported	Study System	Key Finding [Reference]
Neurons (unspecified)	HHV-6	Brains of children with AIDS encephalopathy	Neurons were HHV-6-positive, but less frequently than oligodendrocytes [69]
	HHV-6B	Infant autopsy case of primary encephalitis	HHV-6B-infected neurons, especially in the hippocampus, together with oligodendrocytes and vascular endothelial cells [70]
	HHV-6A and HHV-6B	Differentiated human neural stem cell cultures	Both viruses infected neurons as well as glia and different neurotransmitter phenotypes [67]
Hippocampal neurons	HHV-6 (unspecified)	Human MTLE tissue	HHV-6 DNA and protein reported in hippocampal neurons in MTLE with select studies emphasizing glial localization more strongly [48,62,63]
Purkinje cells	HHV-6A and HHV-6B	Human postmortem cerebellum in mood disorders	Strong tropism for GABAergic Purkinje cells; this is one of the clearest cell-type localization findings in human brain [71]
GABAergic interneurons (molecular layer)	HHV-6A and HHV-6B	Human postmortem cerebellum in mood disorders	Occasional infection of molecular layer GABAergic interneurons was reported [71]
Vascular endothelial cells	HHV-6B	Infant autopsy case of primary encephalitis	HHV-6B-infected vascular endothelial cells together with neurons and oligodendrocytes [70]

**Table 3:** HHV-6A and/or HHV-6B in the CNS: Brain region.

Brain Region	HHV-6 Species Reported	Model System	Key Finding [Reference]
Normal brain tissue (region not specified)	HHV-6 (unspecified)	Human normal brain tissue	Early report of HHV-6 detection in normal human brain tissue [50]
Normal brain tissue and neoplastic nervous tissue (region not specified)	HHV-6A greater than HHV-6B	Human normal and neoplastic brain tissue	HHV-6 DNA detected in both normal and neoplastic nervous tissue; HHV-6A species reported to be found approximately three times more often than HHV-6B [5]
Frontal, temporal, parietal, and occipital lobes; cerebellum	HHV-6A and HHV-6B	Human postmortem brain tissues	Region-by-region PCR-based analyses show HHV-6 DNA across multiple cortical lobes as well as the cerebellum [5]
Hippocampus (astrocytes, TLE resection)	HHV-6 (unspecified)	Human mesial temporal lobe epilepsy tissue	HHV-6 localized in glia MTLE resections; secondary summary notes hippocampal and temporal lobe astrocytes [25]
Hippocampus	HHV-6B	Human MTLE MTS tissue	Active association study linking HHV-6B with MTLE; hippocampus included among tested resected tissues [62,63]
Hippocampus	HHV-6B	Human MTLE MTS tissue	Real-time PCR/RT-PCR study of resected tissue; hippocampus explicitly examined [27]
Hippocampus	HHV-6B (active infection)	Human autopsy after bone marrow stem-cell transplantation	All autopsy samples showed active HHV-6 infection in hippocampus; tropism noted for hippocampal astrocytes [83]
Amygdala	HHV-6B	Human MTLE MTS tissue	Amygdala explicitly examined; HHV-6 DNA burden associated with host-response changes in MTS [27]
Uncus and Amygdala–Uncus (mixed samples)	HHV-6B	Human MTLE MTS tissue	Uncus-containing mixed resection samples explicitly analyzed for HHV-6 DNA/mRNA [27]
Olfactory bulb and Olfactory tract region	HHV-6 (CNS entry)	Human autopsy tissue; additional mouse olfactory cell experiments	Frequency of HHV-6 DNA highest within olfactory bulb/tract region; the data support olfactory route of CNS entry [84]
Cerebellum (posterior cerebellar cortex)	HHV-6A and HHV-6B	Human postmortem mood-disorder brains	Viral DNA and late proteins detected more often in cerebellums with bipolar disorder and major depression than controls [71]
Cerebellum (Purkinje cells)	HHV-6A and HHV-6B	Human postmortem mood-disorder brains	Active HHV-6A/B infection detected in Purkinje cells; HHV-6A infection associated with reduced Purkinje cell size [71]
Cerebellum (astrocytes and microglia)	HHV-6 (in cerebellum)	Human postmortem mood-disorder brains	Staining showed HHV-6-positive astrocytes localized with microglia in cerebellar cortex sections [71]

**Table 4:** HHV-6A and/or HHV-6B in the CNS: Other CNS tissue.

Tissue Type	HHV-6 Species Reported	Model System	Key Finding [Reference]
MS plaques within lesional white matter	HHV-6 (HHV-6B)	Human multiple sclerosis brain tissue	Plaque-associated HHV-6 expression in MS lesions [85]
MS lesions and normal appearing white matter	HHV-6 (unspecified)	Human postmortem multiple sclerosis brain tissue	Early and late HHV-6 gene transcripts detected in MS lesions and normal-appearing white matter [86]
Olfactory pathways (gray and white matter)	HHV-6	Human autopsy olfactory pathway tissue in Unspecified Encephalopathy and controls	HHV-6-positive oligodendrocytes and neurons in gray matter and astrocytes as well as oligodendrocytes, microglia, and endothelium in white matter [68]
Pediatric glioma (glial tumors)	HHV-6A/B antigen reported; exact anatomic region varies by tumor site	Human pediatric brain tumors	HHV-6 antigen detected more often in low-grade gliomas; colocalization with astrocytes reported, but tumor location was not a single defined brain region [75]

**Table 5:** Immune and inflammatory changes following HHV-6 infection: Cytokines.

Immune Marker	HHV-6 Infection Impact	Model System and Cell Type	Key Finding [Reference]
IFN-γ	Increased in acute phase	Patients with primary HHV-6 infection	Supports acute immune activation [99]
IL-2	Increased in acute phase	Patients with primary HHV-6 infection	Supports adaptive immune activation [99]
IL-4	Increased in acute phase	Patients with primary HHV-6 infection	Suggests concurrent Th2 associated response [99]
MCP-1 (CCL2)	Increased in acute phase	Patients with primary HHV-6 infection	Consistent with inflammatory cell recruitment [99]
IL-5	Increased during convalescent phase	Patients with primary HHV-6 infection	Associated with recovery phase immune response [99]
IL-10	Increased in HHV-6A compared to HHV-6B	Differentiated human neural stem cell-derived cultures	Indicates anti-inflammatory response in HHV-6A [67]
TNF-α	Increased in HHV-6B compared to HHV-6A	Differentiated human neural stem cell-derived cultures	Suggests pro-inflammatory response in HHV-6B [67]
IL-6	Trend toward increase with HHV-6A; no significant change with HHV-6B	Differentiated human neural stem cell-derived cultures	Mild, context-dependent inflammatory response [67]
IL-1β	No significant change in dHNSC cultures; increased in HHV-6A-infected microglia	Differentiated human neural stem cell-derived cultures and HHV-6A-infected human microglia	Demonstrates strong cell-type-specific response [2,67]
VEGF-C	Increased with both HHV-6A and HHV-6B	Differentiated human neural stem cell-derived cultures	Suggests vascular and trophic remodeling [67]
Aβ1-42	Significantly increased starting at 3 dpi	HHV-6A-infected human microglial cells	Supports amyloidogenic response [2]
Aβ1-40	No significant induction	HHV-6A-infected human microglial cells	Indicates selective increase in Aβ1-42 [2]
IL-12	Decreased after stimulation	Human macrophages exposed to HHV-6A or HHV-6B	Indicates impaired Th1 cytokine production [100]
TLR signaling output	Impaired downstream cytokine responses despite preserved receptor expression	HHV-6-infected human dendritic cells	Demonstrates immune evasion at signaling level [101]
CCL2, CCL5, CXCL10	Increased	HHV-6A-infected primary brain mixed glial cultures	Promotes neuroinflammatory signaling [102]
CCL5	Increased in brain	HHV-6A infection in CD46 transgenic mouse brain tissue	Validates neuroinflammatory response in vivo [102]

**Table 6:** Immune-related changes following HHV-6 infection: Other molecules.

Immune Marker	HHV-6 Infection Impact	Model System and Cell Type	Key Finding [Reference]
TLR9	Increased in HHV-6A compared to HHV-6B	Differentiated human neural stem cell-derived cultures	Suggests stronger innate sensing in HHV-6A [67]
IGFBP6	Increased with HHV-6A	Differentiated human neural stem cell-derived cultures	Suggests altered growth and stress response signaling [67]
TREM2	Associated with microglial activation after HHV-6A infection	HHV-6A-infected human microglial cells	Supports activated microglial phenotype [2]
ApoE	Associated with microglial activation after HHV-6A infection	HHV-6A-infected human microglial cells	Linked to neurodegeneration-related pathways [2]

**Table 7:** Working hypotheses for HHV-6-induced epilepsy.

Hypothesis	Mechanism	Supporting Evidence
Glutamatergic Hyperexcitability via Primary Infection Mechanisms	HHV-6 may infect glutamatergic cells resulting in abnormally excessive glutamate release either via upregulation of synaptic fusion and release mechanisms or via gross cell lysis (Figure 2). Alternatively, HHV-6 may target GABAergic neurons (or interneurons) reducing inhibitory inputs to excitatory cells (e.g., glutamatergic neurons).	HHV-6 infects glutamatergic neurons [67]. High extracellular glutamate is a major driver of seizure initiation and neuronal injury [151,152,153,154].
Glutamatergic Hyperexcitability via Astrocytic Dysfunction	HHV-6 preferentially infects astrocytes, disrupting glutamate and potassium homeostasis, metabolic support, and the glutamate–glutamine cycle, which promotes gliosis, neuronal injury, and persistent hyperexcitability (Figure 4).	HHV-6-infected astrocytes were detected in epileptogenic tissue. Infected astrocytes exhibit both morphological and functional abnormalities, including dysregulation of EAAT2 and altered glutamine synthetase-related pathways. HHV-6-positive MTLE tissue shows increased GFAP and CCL-2 upon virus infection and neuronal loss [26,27,28,29,118,119,120,155,188,189,190,191].
Neuromodulatory Disruption via Monoamine Neurotransmitter Pathways	HHV-6-associated neuroinflammation can impair dopaminergic, noradrenergic, and serotonergic systems that normally regulate limbic excitability through cAMP/PKA, ERK, DARPP-32, and immediate early gene signaling pathways (Figure 5).	Dopamine (DA), norepinephrine (NE), and serotonin (5-HT) modulate seizure thresholds and epileptiform cell activity. Inflammation due to HHV-6 infection and injury to brain tissue weakens endogenous anticonvulsant systems, thus facilitating seizure propagation [62,67,156,157,158,159,160,161,162,163,164,165,166,167,168,169,170,171,192,193].
Cholinergic Dysfunction	HHV-6 may indirectly disrupt cholinergic regulation of entorhinal hippocampal circuits through glial dysfunction, inflammatory injury, altered muscarinic signaling, disturbed calcium dynamics, and stress-related changes in acetylcholinesterase isoforms (Figure 5).	Cholinergic signaling plays a major role in temporal lobe circuit regulation. Reduced acetylcholine and disrupted cholinergic function have been reported in epilepsy models and viral encephalitis. Although direct evidence for HHV-6 dysregulation of cholinergic neurons remains limited, indirect disruption is biologically plausible [26,29,172,173,174,175,176,177,178,179,180,181,182,183,184,194].
Neuroinflammatory Amplification	HHV-6 may contribute to epileptogenesis through TLR9/MyD88/NF-κB inflammatory signaling, leading to cytokine induction, disrupted glutamate homeostasis, enhanced excitotoxicity, and chronic neuroinflammation (Figure 4 and Figure 5).	HHV-6-infected neurons, astrocytes and oligodendrocytes produce IL-1, TNF-α, IFN-γ, CCL-2, CCL-5, and CXCL-2. NF-κB signaling is linked to HHV-6-associated MTLE, and elevated cytokine production in seizure-associated samples supports an inflammatory contribution to neuronal dysfunction [59,60,65,66,67,82,86,102,195,196,197,198,199,200,201,202,203,204,205,206,207].

## Data Availability

All data presented in this review may be found in prior publications at https://pubmed.ncbi.nlm.nih.gov and https://www.ncbi.nlm.nih.gov/genbank/. URLs accessed multiple times between 1 January 2024 and 15 March 2026.

## Abbreviations

The following abbreviations are used in this manuscript:

5-HT	Serotonin (5-hydroxytryptamine)
5-HT1A/5-HT2C	Serotonin Receptor Subtypes 1A and 2C
Aβ	Amyloid-Beta (Aβ1-40 and Aβ1-42 are specific peptide lengths)
ACh	Acetylcholine
AChE	Acetylcholinesterase
AD	Alzheimer’s Disease
AIDS	Acquired Immunodeficiency Syndrome
AP	Action Potential
BBB	Blood–Brain Barrier
BF	Basal Forebrain (Figure 1)
CA1/CA3	Cornu Ammonis 1/3 (hippocampal subfields)
cAMP	Cyclic Adenosine Monophosphate
CCL-2	Chemokine (C-C motif) Ligand 2 (a.k.a., MCP-1)
CCL-5	Chemokine (C-C motif) Ligand 5 (a.k.a., RANTES)
CD4	Cluster of Differentiation 4 (marker of helper T lymphocytes)
CD46	Cluster of Differentiation factor 46 (HHV-6A primary receptor)
CD134	Cluster of Differentiation 134 (HHV-6B preferred receptor; OX40)
ChAT	Choline Acetyltransferase
ciHHV-6	Chromosomally Integrated HHV-6
CI	Confidence Interval for Statistical Analysis (e.g., 95% CI)
CIS	Clinically Isolated Syndrome (an MS phenotype)
CMV	Cytomegalovirus (a.k.a., HHV-5)
CNS	Central Nervous System
COVID-19	Coronavirus Disease 2019
CRP	C-Reactive Protein
CSF	Cerebrospinal Fluid
Ctx	Cortex (Figure 1)
CXCL-2	Chemokine (C-X-C motif) Ligand 2
D1/D2	Dopamine Receptors Type 1 and Type 2 (D1-like and D2-like families)
DA	Dopamine
DARPP-32	Dopamine- and cAMP-Regulated Phosphoprotein, 32 kDa
DRL/DRR	Direct Repeat Left/Direct Repeat Right (viral genome flanking sequences)
dsDNA	Double-Stranded Deoxyribonucleic Acid
EAAT2	Excitatory Amino Acid Transporter 2 (astrocytic glutamate transporter)
EBV	Epstein–Barr Virus (a.k.a., HHV-4)
EC	Entorhinal Cortex (Figure 1)
EDSS	Expanded Disability Status Scale
eHHV-6	Endogenous HHV-6 (previously known as iciHHV-6)
EEG	Electroencephalogram/Electroencephalography
ERK	Extracellular Signal-Regulated Kinase
GABA	Gamma-Aminobutyric Acid (inhibitory neurotransmitter)
GABAA	GABA Type A Receptor (ionotropic)
GAD67	Glutamate Decarboxylase 67 kDa (GABAergic neuron marker)
gB/gH/gL/gO	Viral Glycoproteins B/H/L/O
GFAP	Glial Fibrillary Acidic Protein (astrocyte marker)
GLT-1	Glutamate Transporter 1 (rodent homolog for EAAT2)
GPe/GPi	Globus Pallidus Externa/Interna (Figure 1)
HBLV	Human B-Lymphotropic Virus (original 1986 designation for HHV-6)
hCD46+	Human CD46-Expressing Cells
HHV	Human Herpesvirus
HHV-1	Human Herpesvirus 1 (a.k.a., HSV-1)
HHV-4	Human Herpesvirus 4 (a.k.a., Epstein–Barr virus, EBV)
HHV-5	Human Herpesvirus 5 (a.k.a., Cytomegalovirus, CMV)
HHV-6A	Human Herpesvirus 6A Roseolovirus
HHV-6B	Human Herpesvirus 6B Roseolovirus
HHV-7	Human Herpesvirus 7
HIV	Human Immunodeficiency Virus
Hpc	Hippocampus (Figure 1)
HSV-1	Herpes Simplex Virus 1 (a.k.a., HHV-1)
HTLV-1	Human T-Lymphotropic Virus 1
ID50	50% Infectious Dose (amount of virus needed to infect 50% of subjects)
iciHHV-6	Inherited Chromosomally Integrated HHV-6 (a.k.a., eHHV-6)
IE1	Immediate-Early 1 (viral gene/protein)
IFN-γ	Interferon Gamma
IL-1/IL-1β	Interleukin-1/Interleukin-1 Beta
IL-8	Interleukin-8
IL-10	Interleukin-10
IL-17A	Interleukin-17A
iPSCs	Induced Pluripotent Stem Cells
KCNQ2/KCNQ3	Potassium Voltage-Gated Channel Subfamily Q, Members 2 and 3
LC	Locus Coeruleus (brainstem noradrenergic nucleus)
M1/M2/M3/M5	Muscarinic Acetylcholine Receptor Subtypes 1, 2, 3, 5
ME/CFS	Myalgic Encephalomyelitis/Chronic Fatigue Syndrome
MEA	Multi-Electrode Array
MOI	Multiplicity of Infection
mRNA	Messenger RNA
MS	Multiple Sclerosis
MTL	Mesial Temporal Lobe
MTLE	Mesial Temporal Lobe Epilepsy
MTS	Mesial Temporal Sclerosis
MyD88	Myeloid Differentiation Primary Response 88 (TLR adaptor protein)
NA	Noradrenergic/Noradrenaline (Figure 5)
NE	Norepinephrine
NF-κB	Nuclear Factor Kappa-Light-Chain-Enhancer of Activated B cells
NKCC1	Na-K-Cl Co-Transporter 1 (regulates neuronal chloride gradient)
NMDA	N-Methyl-D-Aspartate (glutamate receptor subtype)
NPP	Pathological Protein Aggregates
OR	Odds Ratio for Statistical Analyses (e.g., OR = 9.42)
ORF	Open Reading Frame
PDS	Paroxysmal Depolarization Shift
PFC	Prefrontal Cortex (Figure 1)
PKA	Protein Kinase A
PPMS	Primary Progressive Multiple Sclerosis
PPN	Pedunculopontine Nucleus (Figure 1)
qPCR	Quantitative Polymerase Chain Reaction
RRMS	Relapsing–Remitting Multiple Sclerosis
SARS-CoV-2	Severe Acute Respiratory Syndrome Coronavirus 2 (cause of COVID-19)
SCN8A	Sodium Voltage-Gated Channel Alpha Subunit 8 gene
SE	Status Epilepticus
SNc	Substantia Nigra pars Compacta (Figure 1)
SNr	Substantia Nigra pars Reticulata (Figure 1)
SPMS	Secondary Progressive Multiple Sclerosis
STN	Subthalamic Nucleus (Figure 1)
Str	Striatum (Figure 1)
TCID50	50% Tissue Culture Infectious Dose
Th	Thalamus (Figure 1)
TLE	Temporal Lobe Epilepsy
TLR	Toll-Like Receptor (e.g., TLR9)
TNF-α	Tumor Necrosis Factor Alpha
U12/U51/U83/U94	HHV-6 Viral Gene/ORF Designations Referenced in this Paper
VZV	Varicella Zoster Virus
α1/β	Alpha-1/Beta Adrenergic Receptor (Figure 5)
